# The functionality of the gastrointestinal microbiome in non-human animals

**DOI:** 10.1186/s40168-015-0113-6

**Published:** 2015-11-10

**Authors:** Irene Hanning, Sandra Diaz-Sanchez

**Affiliations:** College of Genome Sciences and Technology, University of Tennessee, Knoxville, TN USA; Department of Science, Lincoln International Academy, Managua, Nicaragua; Department of Food Science and Technology, University of Tennessee, Knoxville, TN USA

**Keywords:** Microbiome, Bacteria, Gastrointestinal tract

## Abstract

Due to the significance of the microbiome on human health, much of the current data available regarding microbiome functionality is centered on human medicine. For agriculturally important taxa, the functionality of gastrointestinal bacteria has been studied with the primary goals of improving animal health and production performance. With respect to cattle, the digestive functions of bacteria in cattle are unarguably critical to digestion and positively impact production performance. Conversely, some research suggests that the gastrointestinal microbiome in chickens competes with the host for nutrients and produces toxins that can harm the host resulting in decreased growth efficiency. Concerning many other species including reptiles and cetaceans, some cataloging of fecal bacteria has been conducted, but the functionality within the host remains ambiguous. These taxa could provide interesting gastrointestinal insight into functionality and symbiosis considering the extreme feeding regimes (snakes), highly specialized diets (vampire bats), and living environments (polar bears), which warrants further exploration.

## Introduction

In humans, the numbers of bacterial cells outnumber human tissue cells by 10 to 1. The skin, nasal passages, and gastrointestinal tract are all inhabited with microorganisms, and each location has a specific microbial profile composed of microorganisms best suited to inhabit that niche. Perhaps the most studied of these niches is the gastrointestinal tract (GIT). From Table 1, it is clear that competition, mutualism, and co-habitation all occur within the GIT microbial community as well as provide nutrients and vitamins to the host.

**Table 1:** Examples of microorganisms found within the gastrointestinal tract of the human lower intestine, the substrates utilized by the bacteria, and products made from the substrates

Domain	Taxa	Nitrogen source	Carbon source	Products made	Metabolic potential
Archaea	*Methanobrevibacter smithii*	Ammonium	Carbon dioxide	Methane	Sulfate reducers Polysaccharide fermentation
	*Methanosphaera*	Ammonium	–	Methane	Sulfate reducers
Bacteria	*Actinomyces* sp.	–	d-Glucose, d-mannose, starch, dextrin Glycerol	Formate, acetate, succinate, lactate Various antibiotics	Breakdown and recycle organic compounds
	*Bacteroides* spp.	Ammonium	Starch, cellulose, xylan, pectin, dextran Mucin	Succinate, propionate, acetate, lactate, formate, and malate	Sugar fermentation Increased N source by recycling uric acid waste
	*Bifidobacterium*	Ammonium	Glucose, starch, xylan, pectin, inulin, fructose, galactose	Acetate, lactate, acetone, thiamine, folic acid, nicotinic acid, pyridoxine, biotin Conjugated linoleic acid, vitamins	Vitamin biosynthesis Carbohydrate fermentation
	*Clostridium*	Amino acids	Glucose, starch, mucin, hyaluronic acid	Acetate, butyrate, acetone, butanol	Proteolytic activity
	*Desulfovibrio*	Ammonium	–	Hydrogen sulfide Acetate/ATP	Sulfate reducers
	*Enterococcus*	Collagen, casein, insulin, Hb, fibrogen, gelatin	Glucose	Lactate	Lipoprotein lipase inhibitor Proteolytic activity Polysaccharolytic activity
	*Escherichia coli*	–	Glucose, OS*	Acetic acid, lactic acid, succinic acid, CO_2,_ H_2_	Carbohydrates fermentation
	*Eubacterium*	Amino acids	Glucose, OS*	Lactate, acetate, formate, succinate	Metabolism of carbohydrates Lactate-utilizing bacteria
	*Fusobacterium* sp.	Amino acids	OS*	Butyrate	Proteolytic activity
	*Lactobacillus* sp.	Amino acids	Lactose, OS*	Lactate, acetate	Bile deconjugating activity Folate production
	*Pediococcus* sp.		Lactose, OS*	Lactate	Sugar fermentation Immunomodulatory effects
	*Prevotella* sp.	Dipeptides, amino acids	Pentose, hexose	Acetate, formate, succinate	Mucin glycoprotein degraders
	*Propionibacterium* sp.	Ammonium	Lactose β-Galactosidase	Propionic acid Fatty acids Vitamin B_12_ Branched-chain acids Short-chain fatty acids	Immunomodulatory effects Lipolysis of glycerides Catabolism of amino acids
	*Ruminococcus*	Ammonium	Various carbohydrates	Formate, lactate, succinate	Mucin degraders
	*Staphylococcus*	–	Lactose, OS*	Lactic acid	Glucose fermentation Nitrate reductase and urease activity
	*Streptococcus* sp.	–	Various sugars, soluble starch	Lactate	Polysacharolytic activity
	*Veillonella* sp.	Amino acids	Lactate	Propionate, acetate	Lactate-utilizing bacteria

*OS - Other Solutes

The primary function of the GIT microbes is digestion of ingested food substrates. As shown in Table 2, symbiosis between microbes and their host primarily involves nutrient acquisition. However, beyond digestion, GIT microbes perform other functions that potentially contribute to the overall health status of the host. A well-studied non-digestive function of the gut microbes is the education and regulation of the immune system [1]. The commensal bacteria educate the immune system allowing the host to distinguish commensal and pathogenic bacteria. Commensals can also regulate the immune response in the eukaryotic host cells including the inflammatory cascade via the nuclear factor-kappaB (NF-κB) pathway [2]. NF-κB is a transcriptional regulator that translocates to the nucleus to induce inflammatory cytokines and recruit immune cells. This process only occurs when NF-κB is unbound from IκB. Commensal bacteria block the NF-κB-IκB disassociation and therefore NF-κB cannot enter the nucleus to begin the inflammatory response. Identifying these bacteria and their products could be useful for treating inflammatory-based diseases including inflammatory bowel disease.

**Table 2:** Examples of symbiotic interactions between gut bacteria and the host

Host	Bacteria taxa	Food source	Suggested function	Reference
Stinkbug	*Ishikawaella capsulata*	Plant sap	Nutrient provision Amino acid and vitamin synthesis	[82]
Kissing bug	*Rhodococcus rhodnii*	Blood-based diets	Nutrient provision Growth and development	[83]
Honeybee Leafhopper Caterpillar	*Acetobacteraceae*	Sugar-based diets	Oxidize sugars Acetic acid production	[84, 85]
Drosophila	*Acetobacter* *Lactobacillus*	Sugar-based diets	Activation of insulin hormone signaling	[86]
Goat	*Synergistes jonesii*	Forage	Metabolize toxins	[87, 88]
Reindeer Sheep Elk	*Eubacterium rangiferina*	Forage	Metabolize toxins present in lichen	[89, 90]
Rat	*Enterobacteriaceae* *Bacteroides*	Omnivore	Tannin-resistant bacteria	[91]
Mice	*Helicobacter* spp.	Omnivore	Inducer of CXC chemokine responses in epithelial cell lines	[92]
Tammar wallaby	Unique clades of *Lachnospiraceae, Bacteroides, Gammaproteobacteria*	Herbivore	Plant biomass conversion	[93]
Flying squirrel	Dominated by *Firmicutes*	Herbivore	Degradation of carbohydrates Metabolism of proteins Synthesis of vitamins	[94]
Giant panda	*Clostridium* I and XIVa	Bamboo	Cellulolytic and hemicellulolytic activities	[95]
Zebra	*Lactobacillus equigenerosi*	Herbivore	Plant fermentation	[96]
Horse	*Weissella confusa/cibaria*			
Ostrich	*Fibrobacter succinogenes* *Fibrobacter intestinalis*	Herbivore	Fibrolitic activity	[97]
Grass carp	*Anoxybacillus* *Leuconostoc* *Clostridium* *Actinomyces* *Citrobacter* *Aeromonas*	Herbivore	Cellulolytic activity	[98]
Surgeon fish	*Epulopiscium fishelsoni*	Herbivore	Digestion of herbs and detritus	[99]
Iguanas	*Archaebacteria* (Methanogens)	Herbivore	Fermentation	[100]
American bullfrog tadpoles	*Edwardsiella tarda* *Clostridium*	Herbivore	Carbohydrate fermentation	[101]

Gene regulation by the GIT microbes is not limited to the inflammatory cascade. In fact, Hooper et al. [3] reported that *Bacteroides thetaiotaomicron* modulates 71 intestinal genes involved in various processes including intestinal maturation and nutrient absorption. More recently, Neufeld and Foster [4] implied that the impact of the GIT microbiome reaches beyond the intestinal tract and even can be linked to brain development. A recent study supports this notion by demonstrating that adult germfree mice show an exaggerated stress response versus conventional counterparts [5]. Additionally, it is also well recognized that many gastrointestinal disorders demonstrate a high comorbidity with psychiatric illness [4]. For this reason, emerging work involving germfree mice suggests methods targeting systems outside of the central nervous system are potential treatment options for psychiatric diseases.

A wealth of information regarding the functionality and impact of the gut microbiome on human health has been obtained from clinical studies and experimental models. Currently, studies are applying these methods to non-human animals with bias towards agricultural taxa. Regardless, there are numerous papers available for some species, fewer for others, and no information for some animals. The point of this review is not to provide an exhaustive list of bacterial taxa present in every animal and the functionality of those microbes. Rather, the aim of this review is to point out that some gut microbe functions may be broad and applicable to many animals, while some species may contain unique microbes performing unique functions and to use a few animal species to demonstrate this idea. Deciphering and defining the functionality of the gut microbes can be addressed using various methods and observations.

### Changes, disruption, and absence of the normal microbiome

Assessing and determining the functionality of microbes is not an easy task. Numerous experiments have been undertaken to understand the function of the gut microbiome in different species. These approaches include analyzing animals reared in sterile environments lacking any gut microbiome and transplanting microbiome from one animal to another. Disruptions to the microbiome such as fasting or gastrointestinal infection also provide insight regarding functionality of the commensals. Furthermore, these types of comparisons may be made to enhance the understanding of the symbiotic balance.

#### Germfree

One approach to determine functionality of microbiomes is to compare sets of conventional animals to those without microbiomes. These so-called germfree animals have been documented as early as 1895 by Nuttall and Theirfelder [6] who intended to prove that microbes were not needed for life and were actually harmful by creating germfree guinea pigs. Other experiments including one conducted by Cosendy and Wollman also intended to demonstrate that animals could live without microbes and utilized “germfree” chickens to test their hypothesis [7, 8].

It is clear from early experiments and the newest information that higher organisms can exist without microbes. However, drastic differences are noted in germfree animals. Germfree sea bass (*Centropristis striata*) had reduced mucosal linings due to an absence of microbial stimulation of mucosa production [9]. The intestinal villi of the germfree dog are of the same length as the normal counterpart but are thinner with pointer tips [10]. The lamina propria of germfree mice has a sparse stroma, with few lymphocytes and macrophages, and the Peyer's patches are smaller. In all germfree animals studied, the turnover rate of intestinal epithelium was decreased. These histological changes in the intestine are the result of a reduced interaction with the bacteria that stimulate the immune system and histological development [11].

In addition to histological changes in the intestine, other organs and physiological changes have been noted. The germfree rat has a smaller heart and a cecum that is four to six times larger than rats with normal microbiomes [12]. This change has been explained by an accumulation of substances normally degraded by the microbiome and histological changes of the cecum epithelial cells [13]. In germfree chicks, the body temperature is slightly higher than that of chicks with normal flora [14]. These experiments have demonstrated the complex role that microbiomes play in host development, not only in the gastrointestinal systems but in other organs as well.

#### Fasting

That fasting tends to shift the GIT bacteria populations has been demonstrated in many animals including mice, alligators, pythons, and chickens [15–18]. Furthermore, a feeding/fasting cycle promotes diversity of the microbiome [18]. During fasting, the lack of nutrient availability impacts the intestinal mucin layer, as bacteria with the ability to utilize mucin will degrade it for a nutrient source [19]. Hence, when the host does not provide nutrients to the commensals, the commensals will start to consume the host. This may be because fasting suppresses the host antibacterial defenses [20, 21]. Interestingly, even though the protective barrier of mucin is decreased, bacterial translocation across the epithelia may not increase [21].

Like fasting, hibernation also affects on the microbiome populations. Sonoyama et al. [22] reported that *Clostridium* predominated in both active and hibernating hamsters and populations of *Akkermansia muciniphila*, a mucin degrader, increased in fasting but not during hibernation. An early study of ground squirrels using culturing methods found that there was some reduction in total numbers of viable bacteria in the cecum during hibernation, but that the profile of the microbiota remained stable [23]. However, a more recent study using deep-sequencing methods found that the population profiles did shift. Specifically, hibernation increased populations of *Bacteroidetes* and *Verrucomicrobia*, capable of degrading mucin, and reduced populations of *Firmicutes*, which prefer polysaccharides [24].

Hibernating animals typically have a hypoactive immune system. Because a hypoactive immune system can lead to aberrant bacterial growth and inflammation, understanding how the immune system regulates bacterial populations during hibernation is of great interest. In their studies using hibernating leopard frogs, Gossling et al. [25] tested several hypotheses to understand this regulation, including unique circulating antimicrobials and antibody production, but no conclusive results were able to answer the question. More recently, Dill-McFarland [26] reported that toll-like receptors 4 and 5 (TLR4 and TLR5) are modulated in hibernating ground squirrels. These receptors recognize microbial products and initiate host immune responses focused on inflammation.

Compared with other animal taxa, there is little information about the gastrointestinal microbiome found in reptiles, many of which practice an extreme feeding/fasting cycle. Snakes are very interesting animals because many species have extreme periods of fasting with a time span as long as 1 year between meals [27]. In addition, all snakes ingest and rapidly digest whole prey. These two facts suggest that snakes must be very efficient at nutrient uptake, and this would hold true for the microbiome as well. These extreme feeding regimes in the snake raise the question of whether the microbiome impact on nutrient uptake is more dramatic in the snake or has any affect at all. Peterson et al. [28] aimed to answer this question using African house snakes where the snakes were repeatedly given oral dosages of antibiotics (treatment) or sterile water (control) prior to consuming sterilized mice. Intestinal samples were obtained non-lethally each time prior to feeding and sequencing of these samples was conducted. No differences in energy densities of expelled mice or feces and uric acid as determined by bomb calorimetry were reported. However, the bacteria populations present in the intestinal samples were very different between the two groups. From the data, the authors concluded that the bacterial microbiome had no significant impact on nutrient acquisition.

#### Disruptions and changes

Disruptions in the normal patterns of host microbiome may occur for a variety of reasons. Changes in diet, antibiotic intake, or colonization by pathogens have all been demonstrated to result in a shift in the microbiome populations [29]. In some cases, this shift results in negative health consequences, because the immune system maintains a constant production of antibodies aimed at the normal microbiome pattern that must be adjusted to fit the new bacterial population, and this can be costly in terms of energy expenditures.

Normal changes in the microbiome occur overtime and can be related to factors such as age. For example, the chicken GIT is sterile at hatch but quickly colonized by aerobic *Proteobacteria* and after 12 days is dominated by anaerobic *Firmicutes*. Initially, the Proteobacteria stimulate the histological maturation of the GIT and provide an ideal environment for the *Firmicutes*. The Proteobacteria do well in the immature GIT but are poor competitors and are outcompeted after a mature and anaerobic gut environment is established. The succession of bacteria is also dependent on nutrition and gut bacteria population selection, and establishment can also be selected by feeding specific foods. This was demonstrated in rabbits fed only milk for the first 42 days of life. These animals did not possess cellulolytic bacteria in the cecum and could not digest plant matter [30].

Metamorphosis and the impacts of the process on intestinal populations have been demonstrated in frogs and toads by comparing gut microbes in tadpoles versus adult forms [31]. Tadpoles have more diversity and a microbiome similar to fish, while frog GIT profiles resemble amniotes. The differences are attributed to food preferences and GIT structure. Like amphibians, metamorphosis of the sea lamprey and mosquito results in GIT rearrangement and feeding preferences [32, 33]. In both species, the microbiome in the adult is substantially less diverse than in the young. Specific to the lamprey, the sanguivore form apparently selects for the Aeromonads because this population of bacteria increases from 4 % in the young to 84 % in the parasitic fish. Analysis of these Aeromonads found all species and strains were hemolytic. Like other sanguivores, *Aeromonas hydrophila* was consistently isolated from fecal samples of vampire bats and is thought to be necessary for the digestion of blood [34]. The authors also suggested that acquisition of the bacterium by nursing the young through coprophagy is essential in order to transition to the sanguivore lifestyle.

### Longevity and fecundity

Commensals of the microbiome can be classified as obligate or facultative. The obligate bacteria often have a reduced genome and are dependent on the host and the microbial community for nutrients and other essential compounds. As an example, *Lactobacillus johnsonii* codes for amino acid proteases, peptidases, and phosphotransferase transporters but not for genes necessary for biosynthetic pathways. In addition, *L. johnsonii* contains all of the genes necessary for the synthesis of pyrimidines but not for the synthesis of purines, and therefore, the bacterium must acquire amino acids, peptides, and purine nucleotides as secondary metabolites from other microorganisms or from the human host [35]. Since obligate microbes, like *L. johnsonii*, are dependent on the host for survival, promoting the fitness of the host is advantageous to the symbionts.

In humans, promoting longevity is a leading research focus, and how the microbiome can modulate longevity is of great interest. This research area is just beginning and some animal models have demonstrated a link between the gut microbiome and longevity. It was reported that feeding wild-type soil bacteria *Bacillus* spp*.* to *Caenorhabditis elegans* versus the typical *Escherichia coli* lab strain increases longevity [36]. In *Drosophila*, infection with an avirulent *Wolbachia* extends life-span [37]. The mechanisms by which bacteria can extend life-span have been attributed to host gene regulation of immune factors and cell proliferation and availability of key vitamins and co-factors produced by the microbiome [38, 39].

Unlike humans, the goal in rearing agricultural taxa is not longevity but rather to hasten maturity. Hastening maturation and achieving the shortest rearing period possible to obtain harvest weight reduces production time and, in turn, drives down costs. The rearing period of farm-raised Atlantic salmon has been cut in half by genetic modification. A growth hormone-regulating gene from the Pacific Chinook and promoter from the ocean pout were added to the genome of the Atlantic salmon. This modification allows the fish to grow year-round and achieve market weight in half the time (16 months versus 3 years).

Many agricultural animals do not need to be genetically modified to achieve accelerated growth rate because growth promotion can be obtained through modifying the intestinal microbiome. Delivering probiotic cultures to animals hastens the histological development of the intestinal tract [40, 41]. The mature GIT improves nutrient uptake and increases the growth rate of the animal [40]. Studies in the pig indicate that careful selection of probiotic bacteria is crucial because the bacterial species colonizing the intestinal tract can have a lifelong impact on intestinal health. Shirkey et al. [42] reported differences in the gut histology and immune marker responses dependent on which bacteria initially colonized the gut of the pigs. The group reported that single-strain cultures affected regions of the intestines differently than mixed cultures, and it therefore appears that microbial diversity facilitates healthy maturation of the entire intestinal tract.

It has been argued that the effect of improved growth rate in probiotic-treated animals is temporal, and non-treated counterparts eventually meet the same weight [43]. In fact, some studies have demonstrated that microbes have an overall deleterious impact on growth production in chickens and pigs, where gut metabolism accounts for 20–36 % of the whole body energy expenditure [44–46]. This is due to host-microbiota competition for nutrients, which results in a net energy loss and decreased growth rate. Further, the microbiome stimulates the production of IgA, IgG, and mucin secretion that can cost the animal several hundred grams of protein over a lifetime that is not utilized for growth [47]. Considering the rearing period of a broiler chicken (42 days) and the average final weight (3000 g), a loss of several hundred grams can be significant. Shifts in the gut microbiome may also initiate a costly energy production because different antibodies must be produced [48]. Sub-therapeutic doses of antibiotics delivered in feed can achieve a stable bacterial and antibody production resulting in an increased growth rate and hence their use in some animal production. Regardless of the energy that the microbiome may cost the animal, it is clear that germfree production of agriculture animals to improve production performance is not a realistic practice.

The impact of the microbiome on host fecundity has been demonstrated in a number of insects. Insect studies suggest that oviposition rates are dictated by the volatile compounds produced by the fecal bacteria, and these compounds are olfactory stimulants that are used as positive cues [49]. Similarly, the volatile compounds produced by the gut microbiome can also promote mating aggregations [50]. Rosengaus et al. [51] showed that termites treated with Rifampin reduced the number of nitrogen-fixing bacteria and may have resulted in a loss of amino acids required for oviposition.

In higher animals, there is some evidence of amino acid provisions supplied by the microbiome and an impact on reproduction [52]. But these and other connections between host reproduction and the microbiome are more difficult to discern due to the complexity of higher animal systems. Studies in cows and pigs demonstrate a shift in the GIT microbes during the gestation period, and thesis shifts were inferred to be for fat deposition to support the developing fetus and milk production [53, 54]. It has been suggested that changes in hormones and immune factors during gestation cause subsequent modifications in the microbiome profiles. Thus, it appears that host factors may modulate the microbiome in order to facilitate some physiological needs of reproduction.

### Health and disease

Of all the functions that gastrointestinal bacteria perform, promoting health and preventing disease are likely the most studied. Commensal organisms prevent pathogens from colonizing the host by producing antimicrobial substances (organic acids, bacteriocins) and competing for nutrients and spaces [55]. Pathogens must compete with the commensal bacteria and devise methods to promote infection. For example, *Salmonella typhimurium* induces the inflammatory pathway to reduce the microbial population [56]. Inflammation also provides reactive oxygen species that react with thiosulfate to produce tetrathionate [57]. Tetrathionate can be used by *Salmonella* in the respiratory pathway, and the presence of this compound affords a selective advantage for the pathogen.

In the health promotion framework, the concept of probiotic administration has been extensively explored in numerous taxa to explore the efficacy and benefits of these beneficial cultures. Some have sought probiotic cultures from unusual sources that have unique abilities and might be applied to non-host species. Diaz et al. [58] conducted experiments to determine the ability of the *Lactobacilli* spp*.* isolated from dolphins to inhibit pathogenic growth and stimulate tumor necrosis factor (TNF) production in human monocytoid cells. The authors concluded that many of the strains possessed beneficial probiotic abilities, but whether or not these abilities were active during colonization of the dolphin is still unknown.

Understanding the mechanisms of pathogenesis and how commensal microbes defend the host can lead to therapeutic methods. Conversely, studying the relationship and identification of pathogens can lead to unique methods for control of certain pest insects. One specific example took advantage of ice nucleation to control the mulberry pyralid (*Glyphodes pyloalis*). Ice nucleation refers to the process where insects enhance their supercooling capacity during the winter by eliminating endogenous ice nucleators, accumulating low-molecular-weight polyols and sugars, and synthesizing hemolymph antifreeze proteins to prevent the formation of internal ice crystals that can pierce and damage cells and tissues. In the mulberry pyralid, active bacteria within the gut are known to increase the supercooling points and reduce cold hardiness by expressing an ice nucleation gene. Wantbe et al. [59] colonized the gut of the mulberry pyralid with a strain of *Enterobacter cloacae*, having a transformed ice nucleation gene that led to increased mortality of the insect. A second example involves the medically important triatomine bug that may be colonized by the parasite *Trypanosoma cruzi*. Triatomine bugs were colonized with the mutualistic bacterium *Rhodococcus rhodnii* that was genetically transformed to express an antitrypanosomal peptide effectively preventing colonization and development of the parasite population in the gut of the insect [60].

### Functional dependence for nutrients and digestion

Many herbivores do not produce endogenous cellulases, hemicellulases, and pectinases and as such are dependent on the gut microbiomes for digestion of plant material. Cranial fermenters utilize a rumen in the foregut for fermentation, while caudal fermenters possess a cecum in the large intestines. The location of fermentation has an impact on animal physiology and nutrition. Both types are dependent on the fermentative microbes to extract energy from cellulose. However, unlike caudal fermenters, cranial fermenters cannot utilize hexose sources directly, and these sugars are instead converted to volatile fatty acids in the rumen. Since amino acid absorption takes place in the small intestines, cranial fermenters can utilize the microbes themselves as a source of protein. In fact, in cows, bacterial biomass provides about half the protein requirement for the animal [61]. Conversely, microbial proteins are lost in caudal fermenters because the cecum is located after the small intestines, where amino acids are absorbed. The dependence of the cranial fermenters on microorganisms for digestion is clear when considering the inability to produce healthy germfree adult cows. Germfree calves have been produced and can be sustained for a short time because they feed on sterilized milk, yet adults are nearly impossible to sustain due to the reliance on microorganisms for symbiotic digestion of plant materials.

Although rabbits are caudal fermenters, they can still obtain microbial proteins through cecotrophy. This process is a 2-cycle approach where the first ingestion of plant material is fermented by bacteria in the cecum. Pellets are excreted and ingested and proteins of microbial sources in the pellets are absorbed in the small intestines. Cecotrophes produce two chemically distinct types of feces in order to maximize the extraction of essential nutrients, amino acids, and vitamins from the plants. The impact on the microbial community and the animal due to cecotrophy has been documented. Rabbits prevented from coprophagy have sterile stomachs and may suffer from malnutrition [62].

Marsupial foregut fermenters, or macropods, are cranial fermenters like cows but with anatomical differences (the cow has a four-chambered stomach and the kangaroo only one). Both animals also regurgitate and re-chew food. Rumination in the cow is the key to the digestion process as resalivation of regurgitated feed provides buffers for the rumen that maintain the pH required by the microbiome. In kangaroos, there is little evidence that the regurgitation of food, termed merycism in macropods, is necessary since they eat very slowly and masticate well [63]. Unlike cattle and sheep, kangaroo digestion releases virtually no methane gas during exhalation accomplished by unique microbes converting the hydrogen by-product of fermentation into acetate. These microbes are being sought for use in cattle production to reduce greenhouse gas emission, and some unique microbes of the macropod microbiome have been identified. Pope and co-workers [64] identified bacterial populations present in the tammar wallaby and reported that at the phylum level the *Firmicutes* and *Bacteroides* were the dominant taxa. The group also noted that the majority of the phylotypes were unique and only distantly related to cultured bacteria. Evans et al. [65] reported unidentified methanogens and archaea isolated from the tammar wallaby that were related to bacteria isolated from GITs. The function of these bacteria has not been determined, but genes associated with cell aggregation were identified which they suggested were advantageous for digestion because aggregation facilitates plant biomass digestion.

Many other animals also depend on the gastrointestinal bacteria to digest cellulose. For example, the cellulolytic bacterium *Teredinibacter turnerae* colonizes the Deshayes gland of shipworms, a common group of clams that bore into wooden ships and piers. This bacterium produces cellulases that degrade the wood the clams ingest [66]. The bacterium also fixes nitrogen providing a useable source of nitrogen to the shipworm. The symbiotic dependence for nutrient acquisition in this relationship lacks data because sequencing of the *T. turnerae* genome revealed no deletions in essential genes implying that the bacterium can survive outside the shipworm host [67]. Unlike shipworms, there is plenty of evidence showing that many terrestrial termites are highly dependent on their endosymbionts [68]. In fact, some species die of starvation when the gut microbiome is eradicated with antibiotics [69]. Behaviors including proctodeal feeding suggest termites are aware of their dependence on the microbiome and practice the behavior to ensure transmission of a uniform microbiome among the individuals [70].

A recent study of the American alligator revealed the impacts of evolution on the microbiome for digestive dependence [17]. The authors found a high proportion of *Fusobacterium* in the alligator gut, which is unusual because *Firmicutes* or *Bacteroidetes* typically dominate the gut bacteria populations. For this reason, the authors suggest *Fusobacterium* performs functional roles including development of the digestive organs and nutrient acquisition in the alligator. Furthermore, given that the basal position of *Fusobacterium* on the evolutionary tree and that the American alligator is the least evolved of nearly all animals, the authors suggest that *Fusobacterium* were dominant gut microbes in some prehistoric animals.

### Adaptation to environmental extremes

Marine sponges and tubeworms inhabit a wide distribution of marine environments, but many can be found in locations near hydrothermal vents with pressures of 400 bar (395 atm) and water temperatures up to 60 °C. This environment may also have an acidic pH and is rich in chemicals emitted from the vents including sulfide, hydrogen, and methane. The sponge or tubeworm absorbs chemicals, and microbial symbionts process the chemicals into organic molecules the host needs, while the bacteria gain a stable habitat. The dependence of the tubeworm and sponges on their symbiotic chemoautotrophic bacteria for nutrients has been well established [71]. Studies have confirmed the microbiome of these invertebrates is distinct from the surrounding water [72], and evidence of vertical transmission of endosymbionts exists [73]. Together, data from studies of these animals point to an adaptation of the host to an otherwise uninhabitable environment partly facilitated by the symbiotic bacteria.

Many marine mammals also inhabit extreme environments such as the polar arctic. To date, the few studies examining the gut microbiome of marine mammals living in these extreme environments have identified and cataloged bacteria, but little functionality of these bacteria has been explored. Glad et al. [74] identified the bacteria present in 10 different samples obtained from the rectums of polar bears (*Ursus maritimus; note: polar bears are considered marine mammals and protected as such under marine mammal laws*). Ogawa et al. [75] used cloning techniques to characterize gut bacteria in three different minke whales (*Balaenoptera acutorostrata*). These studied animals are carnivorous, feeding primarily on fish (minke whales) or seals (polar bears), and thus, the identification of bacteria including *Bacteroidetes* and *Firmicutes* is not surprising. Although primarily characteristic in nature, the distribution and abundance of the bacteria in these studies may provide clues for functionality. *Firmicutes* were the dominant taxa in nearly all the samples, and these bacteria have an increased capacity to stimulate host energy storage over other phyla [76]. These animals rely heavily on fat deposition for survival not only for energy storage but also to maintain body temperature. Thus, it is possible that some animals living in the polar extremes rely on their gut microbes to facilitate efficient fat storage needed for survival.

### Functionality revelations by comparative analysis

Intra-species comparisons of the metagenomes of individuals have revealed the functionality of some digestive communities. Qu et al. [77] used a chicken model to show that even though two individuals may have marked differences in microbiomes, the functional metagenome can be the same. They concluded that in their samples, bacterial taxa replaced other taxa without changing the overall function of the microbiome. Conversely, comparison of rumen samples among individuals showed marked differences in microbiomes that did result in differences in the overall functional microbiome [78]. The conflicting results in the two studies may be attributed to diet and digestive tract physiology.

Comparing phylogenetically divergent animals can also provide a better understanding of how microbiomes function and evolve and are shaped by factors such as diet and physiology. Although the cow and termite are both heavily dependent on bacteria for celluloytic digestion, diet was the main factor that shaped the microbiomes in these animals [78]. Comparison of the cow and hoatzin (a foregut fermenting bird) revealed that very similar microbial compositions and organ digestive functions dictated microbial profiles rather than phylogeny [79]. Interestingly, comparative genomic studies have demonstrated that the bacterial profile in swine and humans are similar and harbor many of the same phylogenetic groups primarily due to the similar gastrointestinal systems [80]. However, comparative metagenomic analysis of the pig, chicken ceca, cow rumen, and human fecal microbiomes showed that the metagenomes of the pig aligned more closely with the cow and chicken than with the human microbiome [81]. The results imply that agricultural taxa possess bacterial metagenomes that have been selected as a result of a very consistent and narrowly defined diet and rearing environment.

## Conclusions

Studies of the gastrointestinal tract of animals have produced a wealth of information and revealed that populations are contingent on a number of factors including diet, habitat, and taxonomy. Although animals may be very different evolutionarily, they may have very similar microbes based on specific factors. Mosquitoes, leeches, sea lamprey, and bats are all sanguivores but have very different habitats and are quite distant on the evolutionary tree. However, the digestive tract is dominated by *Aeromonas* spp. in all these animals and is most likely for digestion of blood. Similarly, herbivorous fish, cows, and insect termites have similar cellulolytic microbiota with cows.

Some bacteria species are present in the microbiomes of a wide variety of animal taxa. Conversely, there are unique microbes performing unique functions in a very narrow group or species. Identifying, harvesting, and transplanting these microbes to other animal systems have been sought to perform the same function in non-host species. This concept has been applied to the use of kangaroo intestinal bacteria in the cattle rumen to reduce methane emission. Like other cross-species transplants, application of kangaroo bacteria in the cattle has failed even though the animal system is quite similar. Even at the breed level, it appears that genetics has a large impact on microbial population and function. Transplants of the same microbe into two different chicken breeds resulted in faster weight gain in only one of the breeds. Bacterial transplant failure may be the result of host specificity obtained through co-evolution. This co-evolution has selected the functional roles of the microbiome in a specific host. Thus, understanding the functional roles of microbes and how they interact with the host might further our ability to successfully transplant microbes.
